# Cytogenetic comparison between two allopatric populations of *Astyanax altiparanae* Garutti et Britski, 2000 (Teleostei, Characidae), with emphasis on the localization of 18S and 5S rDNA

**DOI:** 10.3897/CompCytogen.v5i3.1235

**Published:** 2011-08-24

**Authors:** Rosiley Berton Pacheco, Renata da Rosa, Lucia Giuliano-Caetano, Horácio Ferreira Júlio Jr., Ana Lúcia Dias

**Affiliations:** 1 Departamento de Biologia Geral, CCB, Universidade Estadual de Londrina, Londrina, PR, Brazil; 2 Departamento de Biologia Celular e Genética, Universidade Estadual de Maringá, Maringá, PR, Brazil

**Keywords:** Teleostei Characidae, 18S rDNA, 5S rDNA, FISH, karyotypic formula, NORs

## Abstract

Two populations of *Astyanax altiparanae* (Garutti & Britski, 2000) of the Água dos Patos stream/SP and lake Igapó/PR were analyzed. All individuals showed 2n = 50, however, different karyotypic formulae were observed. The population of the Água dos Patos stream showed 8m +24sm+6st+12a (NF=88) and the population of lake Igapó, 8m+28sm+4st+10a (NF=90). Nucleolus organizing regions (AgNORs) were observed in the terminal position on the short and long arm of different chromosomes of both populations, showing a variation from 3 to 4 chromosomes. Fluorescent *in situ* hybridization (FISH) using 18S rDNA probes revealed only one pair of chromosomes with fluorescent signals in the terminal site on the short arm in the Igapó lake population, while the population of Água dos Patos stream showed 4 fluorescence terminal signals, characterizing a system of simple and multiple NORs, respectively. 5S rDNA fluorescent signals were detected in the interstitial position of a pair of chromosomes in the two studied populations. Some AgNOR sites revealed to be GC-rich when stained with Chromomycin A3 (CMA3), however, AT positive regions were not observed. The data obtained show that, despite the conservation of the diploid number and location of 5S DNAr, differences in both the distribution of 18S rDNA and karyotypic formula among the populations were found, thus corroborating the existing data on chromosome variability in *Astyanax altiparanae* that can be significant for cytotaxonomy in this group.

## Introduction

*Astyanax* Baird & Girard, 1854, the most common and diversified genus within the family Characidae, has a wide distribution in the Neotropical Region. Due to lack of evidence of monophyly, the genus *Astyanax* is thought to belong to the Incertae sedis group (Lima et al. 2003). Moreover, the presence of several similarities among the species of this genus allows several species to be considered as a compound from a taxonomic viewpoint (Garutti and Britski 2000).

In *Astyanax altiparanae* (Garutti & Britski, 2000) from the upper Parana river basin, previously identified as *Astyanax bimaculatus* (Linnaeus, 1758), all cytogenetic studies accomplished so far reported the occurrence of 2n = 50, with differences in the karyotypic formula among the analyzed populations (Ferreira Neto et al. 2009), which can be explained by the occurrence of chromosome rearrangements, such as pericentric inversions (Domingues et al. 2007).

Besides the differences in karyotypic formula, the nucleolus organizer regions in this species also vary in relation to number and position, as observed by Fernandes and Martins-Santos (2006a), Domingues et al. (2007), Ferreira Neto et al. (2009). However, the same authors found evidence for conservation in relation to the location and number of fluorescent signals of 5S rDNA sites located in the interstitial region of one chromosome pair.

In view of the great chromosome variation observed by other authors in the genus *Astyanax*, the objective of the present work was to characterize the karyotypes of two populations of *Astyanax altiparanae*, with emphasis on the location of 18S and 5S DNAr sites, and compare them with data contained in the literature, in an endeavor toward a better understanding of chromosome evolution within this fish group.

## Material and methods

**Figure 1.**
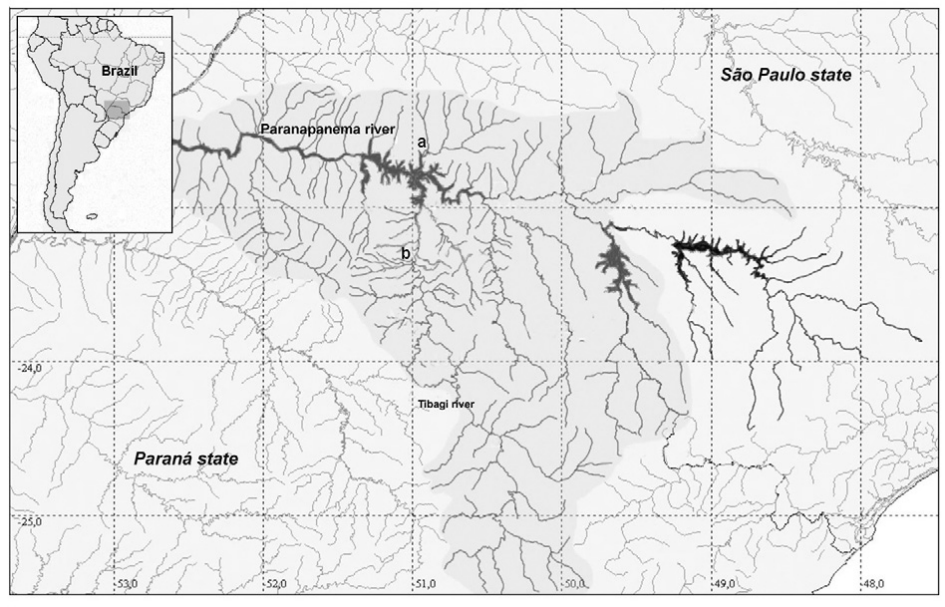
Collection sites of studied specimens. Map of Brazil showing the Paraná and São Paulo states in the selected area (left side). Hydrographic map showing the Paranapanema and Tibagi rivers. In (a) Água dos Patos stream and (b) Igapó lake.

Two populations of *Astyanax altiparanae* were cytogenetically analyzed: twelve specimens (3 males and 9 females) from Água dos Patos stream (22°41'17.7"S; 51° 05'23.9"W ), municipality of Iêpe/SP and sixteen specimens (9 males and 7 females) from Igapó lake (23°19'09.38"S; 51°11'44.72"W ), municipality of Londrina/PR (Fig. 1). Specimens were deposited in the Museum of Zoology of the Universidade Estadual de Londrina (MZUEL). The samples were collected with the permission of Instituto Brasileiro do Meio Ambiente e dos Recursos Naturais Renováveis (IBAMA), protocol number 11399-1. This study was approved by the ethics committee of our institution and meets all requirements of the Brazilian environmental laws.

**Conventional staining.** The specimens were sacrificed after being anesthetized with a solution of benzocaine. Metaphase chromosomes were obtained from kidney cells according to the air drying technique (Bertollo et al. 1978) and stained with 5% Giemsa in phosphate buffer (pH 6.8) The chromosomes were organized as metacentric (m), submetacentric (sm), subtelocentric (st) and acrocentric (a) for the preparation of a karyogram. Metacentric, submetacentric, and subtelocentric chromosomes were considered biarmed and acrocentric uniarmed for determination of the fundamental number (FN) according to Levan et al. (1964).

Fluorescent in situ hybridization (FISH). The *in situ* hybridization procedure was performed according to Swarça et al. (2001). The 18S rDNA probe of *Prochilodus argenteus* Agassiz, 1829 (Hatanaka and Galetti Jr. 2004) and 5S rDNA of *Leporinus elongatus* Valenciennes, 1850 (Martins and Galetti Jr. 1999) were labeled with biotin-14-dATP by nick translation and used as probes. Slides were treated with 30 µl of hybridization mixture (stringency of 70%) containing 100 ng of labeled probe (4 µl), 50% formamide (15 µl), 50% polyethylene glycol (6 µl), 20´ SSC (3 µl), 100 ng of calf thymus DNA (1 µl) and 10% SDS (1 µl). The probe was denatured at 90°C for 10 min, and hybridization was performed overnight at 37°C in a humidified chamber. Post-hybridization washes were carried out in 2´ SSC, 20% formamide in 0.1´ SSC, 0.1´ SSC and 4´ SSC/0.2% Tween 20, all at 42°C. The probe was detected with a solution of 5% BSA and FITC-conjugated avidin (50:0.5, v:v). The post-detection washes were performed in 4´ SSC/0.2% Tween 20 at room temperature. Slides were mounted with 25 µl of a medium composed of 23 ml of DABCO solution (1,4-diaza- bicyclo (2.2.2)-octane (2,3%), 20 mM Tris HCl, pH 8.0, (2%) and glycerol (90%), in distilled water), 1 ml of 2 mg/ml DAPI and 1 ml of 50 mM MgCl2.

Chromosome banding. Active nucleolus organizer regions (NORs) were detected by silver nitrate staining (Howell and Black 1980). The GC- and AT-rich bands were detected with chromomycin A3 (CMA3) and 4’-6-diamino-2-phenylindole (DAPI), respectively, according to Schweizer (1976). The slides were stained with 0.5 mg/mL CMA3 for 1 h, washed in distilled water and sequentially stained with 2 µg/ml DAPI for 15 min. Slides were mounted with a medium composed of glycerol/McIlvaine buffer (pH 7.0) 1:1, plus 2.5 mM MgCl2.

All the images were acquired with a Leica DM 4500 B microscope equipped with a DFC 300FX camera and Leica IM50 4.0 software, and optimized for best constrast and brightness with iGrafx Image software.

## Results

**Figure 2.**
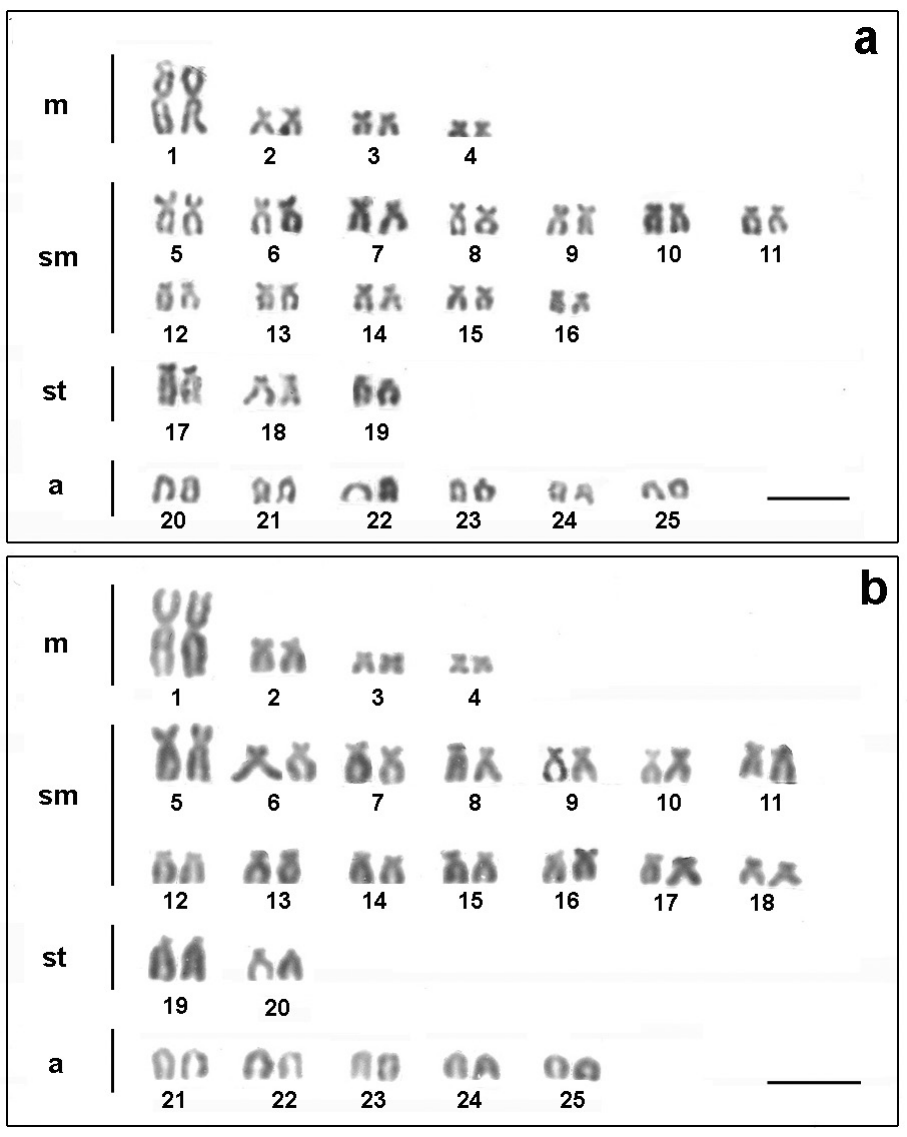
Karyotypes of *Astyanax altiparanae* after conventional Giemsa staining **a** Água dos Patos stream **b** Igapó lake. Bar= 5μm.

The two populations of *Astyanax altiparanae* showed 2n = 50, however, different karyotypic formula were evidenced. The population of the Água dos Patos stream showed 8m+24sm+6st+12a (NF=88) (Fig. 2a) and the population of Igapó lake 8m+ 28sm+ 4st+10a (NF=90) (Fig. 2b).

**Figure 3.**
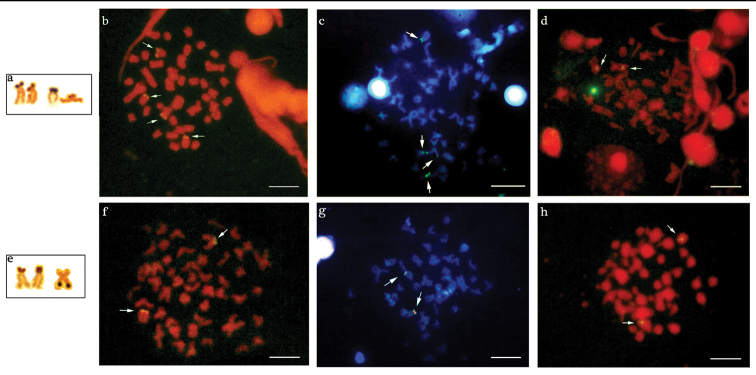
Chromosomes of *Astyanax altiparanae* bearing AgNORs sites: **a** Água dos Patos stream e Igapó lake. Metaphase chromosomes with the arrows showing the 18S rDNA, CMA3/DAPI and 5S rDNA sites in Astyanax altiparanae, respectively: **b, c, d** Água dos Patos stream **f, g, h** Igapó lake. Bar = 5μm.

Multiple NORs sites were detected in the two populations by silver nitrate impregnation, revealing inter- and intra-individual variations. The population of the Água dos Patos stream showes 2 to 4 AgNORs on the short arm, in two equal medium-sized subtelocentric chromosomes, one of which revealed size heteromorphism observed in all metaphases (Fig. 3a).

One to three AgNORs were detected in the population of Igapó lake: a medium-sized submetacentric chromosome with markings on the long arm and also a medium-sized subtelocentric chromosome with signal on the short arm, presenting a small size heteromorphism. The latter was observed in almost all metaphases (Fig. 3e).

The population of the Água dos Patos stream showed 4 markings on the short arm of two medium-sized subtelocentric chromosome pairs after fluorescent in situ hybridization (FISH) with 18S rDNA probe (Fig. 3b). In the population of Igapó lake, only one pair of medium-sized subtelocentric chromosome presenting fluorescent signals on the short arm was detected (Fig. 3f). Both populations exhibited one pair of chromosomes, bearers of the 5S DNAr sites, in the interstitial position (Fig. 3d and h).

In the population of the Água dos Patos stream, 2 to 4 chromosomes with CMA3+ terminal blocks were detected: a medium-sized subtelocentric pair with signals of size heteromorphism on the short arm, most frequently visualized in the metaphases; a small-sized acrocentric chromosome with a marking on the short arm; and a medium-sized subtelocentric chromosome with a signal on the long arm (Fig. 3c). In the population of Igapó lake, CMA3 markings were detected on the short arm of only one pair of medium-sized subtelocentric chromosomes, which disclosed size heteromorphism (Fig. 3g).

The treatment of the chromosomal preparations of the two populations with DAPI showed a homogeneous staining region, and no regions rich in AT base pairs were detected, as can be seen through the superposition of these fluorochrome, as shown in Fig. 3c-g.

## Discussion

Cytogenetic studies in *Astyanax altiparanae* from the Água dos Patos stream and Igapó lake revealed a conserved diploid number that has been observed within all the analyzed populations of this species (Table 1) so far. However, differences in the karyotypic formula were found in some of these populations, including the one observed in the present study, probably due to occurrence of chromosomal rearrangements, such as pericentric inversions, thus revealing a variability in the karyotypic macrostructure among the species of this group of fish.

**Table 1.** Cytogenetic data of different populations of *Astyanax altiparanae*. FN: fundamental number; m: metacentric; sm: submetacentric; st: subtelocentric; a: acrocentric; AgNORs: nucleolar organizer regions; Ref: Reference; PR: Paraná; SP: São Paulo

*Locality*	*2n*	*FN*	*Chromosome formulae*	*AgNORs*	*CMA3*	*18S/28S*	*5S*	*Ref.*
Mogi Guaçu river/SP	50	88	10m+24sm+4st+12a					1
	50	92	6m+24sm+12st+8a	1 a 5				2
Paranapanema river/SP	50	88	10m+22sm+6st+12a					3
Tibagi river/Sertanópolis/PR	50	90	10m+22sm+8st+10a	2 a 5	5			4
Tibagi river/Limoeiro/PR	50	86	6m+22sm+8st+14a	2 a 5	5			4
Tibagi river/Limoeiro/PR	50	88	10m+22sm+6st+12a	2 a 5	5			4
Couro de Boi river/PR	50	88	8m+20sm+10st+12a	1 a 4	6			4
Três Bocas stream/PR	50	92	10m+28sm+4st+8a	1 a 6	8			4
Claro river/PR	50	90	10m+26sm+4st+10a	1 a 4	6			5
		88	10m+24sm+4st+12a	1 a 4	6			5
		86	10m+22sm+4st+14a	1 a 4	6			5
Paraná river/PR	50	82	32m/sm+18st/a	3		4	2	6
Índios river/PR	50	90	6m+30sm+4st+10a	10	7			7
Paraná river/PR	50	88	6m+26sm+6st+12a	2	5	4	2	7,9
Tibagi river upper/PR	50	92	6m+28sm+8st+8a	4	7	7	2	8
Iguaçu river upper/PR	50	94	6m+30sm+8st+6a	2	4	2	2	8
Keçaba brook/PR	50	88	6m+26sm+6st+12a	3		7	2	9
Tatupeba brook/PR	50	88	6m+26sm+6st+12a	3		4	2	9
Maringá stream/PR	50	88	6m+26sm+6st+12a	1		4	2	9
	50	88	10m+22sm+6st+12a	2 a 3				10
Iguaçu river/PR	50	92	10m+26sm+6st+8a	2 a 5				10
Monjolinho river/SP	50	90	8m+20sm+12st+10a	2		2	2	11
Água dos Patos stream/SP	50	88	8m+24sm+6st+12a	2 a 4	2 a 4	4	2	12
Igapó lake/PR	50	90	8m+28sm+4st+10a	1 a 3	2	2	2	12

References:

**1** Morelli et al. 1983

**2** Paganelli 1990

**3** Daniel-Silva and Almeida-Toledo 2001

**4** Pacheco 2001

**5** Pacheco et al. 2001

**6** Almeida-Toledo et al. 2002

**7** Fernandes and Martins-Santos 2004

**8** Domingues et al. 2007

**9** Fernandes and Martins-Santos 2006a

**10** Abelini 2007

**11** Peres et al. 2008

**12** Present study.

The great variability in the karyotypic macrostructure is also reflected in other chromosome marks of *Astyanax altiparanae*. Variability in AgNOR sites with respect to the number, location and types of chromosomes bearers of such sites is frequently evidenced in this species (Table 1), as corroborated by the present study. Some authors consider that such variations can be ascribed to chromosomal rearrangements and transfer of ribosomic sites (Fernandes and Martins-Santos 2006a, Peres et al. 2008) However, in several cases, transposition events have been held liable for that variability of NORs in the genoma of these animals (Mantovani et al. 2000).

It is worth noting that an AgNORs pair was the most frequently found in the chromosome preparations of the populations analyzed herein. It can be considered a main pair with NOR always active, together with secondary sites, as observed by Pazza et al. (2006) in *Astyanax fasciatus* (Cuvier, 1819).

After FISH with 18S rDNA probe, *Astyanax altiparanae* of Água dos Patos stream showed two chromosome pairs with fluorescent signals on the short arms, coinciding with the sites detected by silver impregnation. The population of Igapó lake, however, showed only one chromosome pair with fluorescent signals on the short arm, coinciding with a pair frequently identified by silver nitrate, thus characterizing a system of simple NORs. The other markings, which had not been identified by FISH, but were observed in this population after the impregnation with silver nitrate, are probably heterochromatic sites with acid proteins that have affinity to silver. Despite the fact that multiple NORs are a common condition among *Astyanax altiparanae* (Table 1), Domingues et al. (2007) and Ferreira Neto et al. (2009) also found a system of simple NORs confirmed by FISH in different populations of this species.

Multiple 18S rDNA sites were also found among other species of the genus *Astyanax*, such as *Astyanax scabripinnis* (Jenyns, 1842) (Souza et al. 2001, Mantovani et al. 2005, Fernandes and Martins-Santos 2006b) and *Astyanax fasciatus* (Pazza et al. 2006), which are, therefore, a characteristic of this group of fish.

The CMA3+ sites of *Astyanax altiparanae* of Igapó lake and Água dos Patos stream were consistent with those marked by silver, however, the AgNORs not detected through CMA3 in the individuals of Igapó lake may be very small and not detectable by this fluorochrome, or else not all NORs are rich in GC, as suggested by Artoni et al. (1999).

The Ag-NOR heteromorphism observed in the two populations was not seen by FISH, thence, it should be related to the expression of the genes or be ascribable to a larger amount of heterochromatin that insert in 18S DNAr cistrons, once it was detected by CMA3 and by the impregnation with silver nitrate.

After staining with DAPI, markings were not observed in the chromosomes of the two populations of *Astyanax altiparanae*, which, therefore, did not possess any region rich in AT bases. Rosa et al. (2009) used CMA3 and DAPI fluorochromes in chromosome preparations of *Astyanax laticeps* (Cope, 1894) and observed the occurrence of NORs rich in GC and poor in AT bases, respectively.

In all the populations of *Astyanax altiparanae*, the5S DNAr sites were located interstitially in one chromosome pair, demonstrating a high stability of those sites. This corroborates the data on other populations of *Astyanax altiparanae* (Table 1) and of other species of the genus *Astyanax* (Almeida-Toledo et al. 2002, Fernandes and Martins-Santos 2006b, Pazza et al. 2006). The conservation of this pattern can be attributed to the interstitial location of those sites in the chromosomes, whereby the 5S DNAr is protected from the dispersion events that may occur with 45S DNAr, as proposed by Martins and Galetti Jr. (2001).

According to Ferreira Neto et al. (2009), the apparent karyotypic similarity among the populations of *Astyanax altiparanae* strongly suggests an intimate relationship among them. However, the small karyotypic variations detected indicate some evolutionary divergence, probably due to restrictions on gene flow. The data obtained confirm the occurrence of similarity in relation to diploid number and 5S rDNA location, however, differences in the karyotypic macrostructure and in the distribution of 18S rDNA sites are found among the populations. Thence, from the results obtained in this work we corroborate the existing data and, once again, confirm the great chromosome variability of *Astyanax altiparanae* that can be significant for cytotaxonomy in this group.
